# Structure-Function of Falcipains: Malarial Cysteine Proteases

**DOI:** 10.1155/2012/345195

**Published:** 2012-02-19

**Authors:** Kailash C. Pandey, Rajnikant Dixit

**Affiliations:** Host-Parasite Interaction Biology Group, National Institute of Malaria Research, Indian Council of Medical Research, Sector-8, Dwarka, New Delhi 110 077, India

## Abstract

Evidence indicates that cysteine proteases play essential role in malaria parasites; therefore an obvious area of investigation is the inhibition of these enzymes to treat malaria. Studies with cysteine protease inhibitors and manipulating cysteine proteases genes have suggested a role for cysteine proteases in hemoglobin hydrolysis. The best characterized *Plasmodium* cysteine proteases are falcipains, which are papain family enzymes. Falcipain-2 and falcipain-3 are major hemoglobinases of *P. falciparum*. Structural and functional analysis of falcipains showed that they have unique domains including a refolding domain and a hemoglobin binding domain. Overall, the complexes of falcipain-2 and falcipain-3 with small and macromolecular inhibitors provide structural insight to facilitate the design or modification of effective drug treatment against malaria. Drug development targeting falcipains should be aided by a strong foundation of biochemical and structural studies.

## 1. Introduction

Malaria caused by *Plasmodium falciparum *is responsible for 880,000 deaths per year worldwide [1]. The regions where malaria is endemic include Africa, Asia, and South America [2]. A number of drugs are currently available to treat malaria [3]; however, treatment is becoming complicated by drug resistance, toxicity, and high cost. Recently, drug resistance against the new effective drug, artemisinin, is also emerging [4, 5], and we need new effective drugs to treat malaria. Therefore, the development of other classes of effective antimalarials, especially compounds that act against novel biochemical targets, is required. To develop such compounds, it is very important to characterize the structural and biochemical features of new drug targets.

Among potential new targets for antimalarial chemotherapy are *Plasmodium* proteases. Proteases are druggable targets, and at present protease inhibitors are now licensed as well as in clinical development to treat different diseases for example osteoporosis, diabetes, cancer, hypertension, and infectious diseases [6–9].

Recent advances, including the sequencing of *Plasmodium *genomes (http://www.plasmodb.org/) and development of new tools for manipulating *Plasmodium* genes [10], have improved our understanding of the cysteine proteases of parasites. Therefore, given the importance of the cysteine proteases, this paper will focus on structural-functional relationship of falcipains, major cysteine proteases of *P. falciparum.*

**Figure 1:**
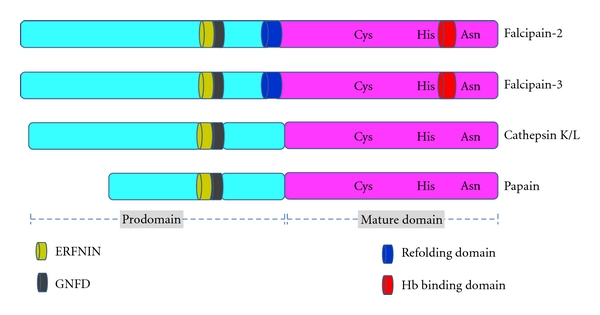
Falcipains have unique features. Falcipains are papain family cysteine proteases, active site residues (Cys, His, Asn) are conserved within the papain family, but falcipains have a unique N-terminal extension acting as a refolding domain, and a C-terminal insert as a hemoglobin (Hb) binding domain. Prodomain has ERFNIN and GNFD motifs, conserved in falcipains, papain, and cathepsin.

In cases of malaria, the parasite relies on human hemoglobin hydrolysis to supply amino acids for protein synthesis and to maintain osmotic stability [11, 12]. Cysteine proteases are involved in hemoglobin hydrolysis and have been validated as promising drug targets [13, 14]. In a recent report, Ch'ng et al., using cysteine protease inhibitors, demonstrate that clan CA cysteine proteases of *P. falciparum* are also involved in chloroquine-mediated programmed cell death [15]. The best characterized *Plasmodium* cysteine proteases are the falcipains, papain family (clan CA) enzymes (Figure 1).

Among the four *P. falciparum* cysteine proteases, falcipain-2 and falcipain-3 appear to be the principal food vacuolar hemoglobinases [16–18]. When the falcipain-2 gene is disrupted, undegraded hemoglobin accumulates in the food vacuole, confirming that this enzyme participates in haemoglobin hydrolysis [17]. However, disruption of falcipain-3 could not be achieved, but the gene was readily replaced with a tagged functional copy, indicating that falcipain-3 is essential for erythrocytic parasites [18]. Falcipain-2 and falcipain-3 share 67% sequence identity and contribute more or less equally to the digestion of hemoglobin in the food vacuole. Comparing with other papain family proteases, falcipains contain unique domains (Figure 1). Specifically, this paper will survey available information on structure and function of different domains of falcipain-2 and falcipain-3 and their interaction with inhibitors.

## 2. Domains of Falcipains

### 2.1. The N-Terminal Part of the Prodomain Is Required for Trafficking of Falcipains to the Food Vacuole

**Figure 2:**
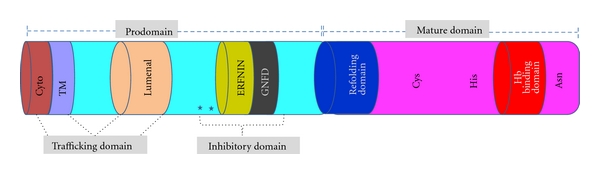
Domains of falcipain-2: schematic diagram showing different domains of falcipain-2. Prodomain is made up of cytoplasmic, transmembrane, luminal, and inhibitory domains. The mature domain has refolding domain, hemoglobin (Hb) binding domain, and catalytic triad residues (Cys, His, Asn). The conserved residues (Phe^165^, Phe^168^) present in inhibitory domain are labeled with asterisks.

Falcipains are major cysteine protease, named due to the function of a catalytic cysteine, which mediates protein hydrolysis via nucleophilic attack on the carbonyl carbon of a susceptible peptide bond [19]. Falcipains have two main domains, the prodomain and the mature domain. The prodomain is further divided into different small domains. At the N-terminus of the prodomain, the first 35 amino acids are cytosolic, followed by a 20 amino acid transmembrane domain and a 188 amino acid lumenal domain. The inhibitory domain is present in the C-terminal part of the prodomain (Figure 2).

Cysteine proteases of the parasite hydrolyze hemoglobin in the acidic food vacuole [20, 21]. Recently, it has been demonstrated that prodomain has unique motifs which are responsible for targeting the falcipains. Using transfection technology and constructing different chimeras with portions of the N-terminal part of prodomain fused to green fluorescent protein, it has been shown that the prodomains of falcipain-2 and falcipain-3 were sufficient to target green fluorescent protein to the food vacuole. The transmembrane domains of falcipain-2 and falcipain-3 are required for entry into the ER. The absence of transmembrane domains led to localization of GFP chimeras to the cytoplasm. Once enzymes are in the ER, using the signal from transmembrane domain, the proteins appear to traffic to the plasma membrane. At the plasma membrane falcipain-2 and falcipain-3 appear to be endocytosed in a process requiring the presence of both cytoplasmic and luminal prodomain trafficking motifs, followed by vesicular transport to the food vacuole [22].

**Figure 3:**
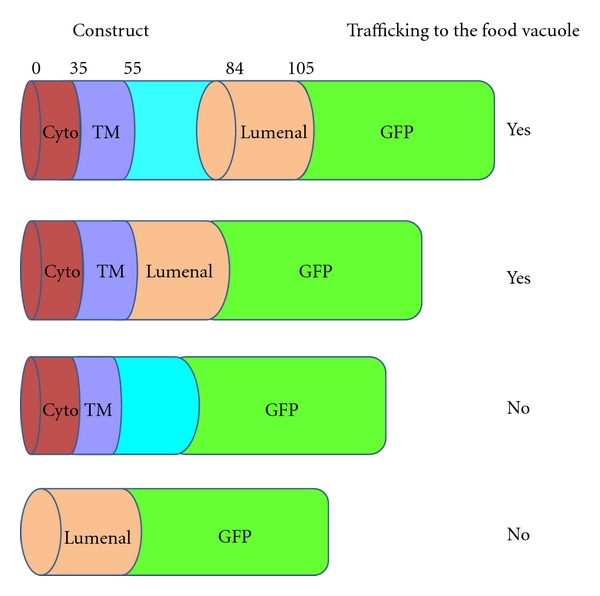
Schematic representation of the profalcipain-2-GFP constructs and their localization. Cytoplasmic (Cyto), transmembrane (TM), and lumenal portions of the prodomain are labeled. Green fluorescent protein is fused to study the trafficking of enzyme.

 In summary, serial truncation and deletion studies showed that both a 20-amino acid stretch of the lumenal portion and a 10-amino acid stretch of the cytoplasmic portion of the enzymes were essential for food vacuolar trafficking (Figure 3) [22].

### 2.2. The C-Terminal Part of the Prodomain Is Required for Inhibiting the Mature Domain

Like many other proteases, falcipains are synthesized as a zymogen, and the prodomain inhibits the activity of the mature enzyme [23]. To investigate how the prodomain regulates the activity of falcipain-2, Pandey et al. expressed constructs encoding different portions of the prodomain and tested their ability to inhibit recombinant mature falcipain-2. It has been found that a C-terminal segment (Leu^155^–Asp^243^) of the prodomain, including two motifs (ERFNIN and GNFD) that are also conserved in cathepsin L subfamily proteases, mediates prodomain inhibitory activity [24].

Earlier work with other cathepsin L subfamily proteases suggests key roles for conserved hydrophobic amino acids (phenylalanine and tryptophan) as well as the ERFNIN and GNFD motifs in maintaining prodomain structure [30]. Later, Pandey et al. explored the roles of conserved motifs in maintaining prodomain structure by circular dichroism analysis. Secondary structure was seen in a fragment with potent inhibitory activity (Leu^155^–Asp^243^), but not in two larger constructs that lacked any sequence downstream of the ERFNIN and GNFD motifs or in a peptide spanning the ERFNIN and GNFD motifs [24]. These results indicate that, together with ERFNIN and GNFD motifs, an upstream region including two conserved Phe* residues is required for proper folding or maintenance of secondary structure of the prodomain (Figure 2).

**Figure 4:**
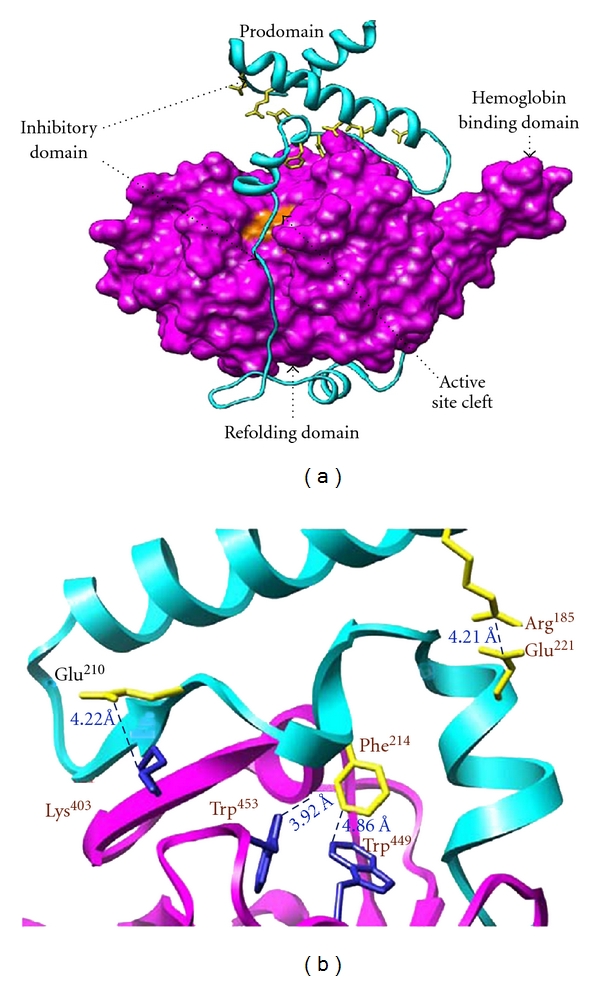
(a) Structure of profalcipain-2: The mature domain (magenta) of falcipain-2 was solved by X-ray diffraction. The active site (catalytic triad residues in orange), refolding domain, and hemoglobin domain are labeled [25]. The prodomain (cyan) modeling was done based on procathepsin L and K [24]. The prodomain runs up the face of the mature enzyme (purple) before forming *α* helices containing the conserved ERFNIN and GNFD motifs (yellow). Since there is no homology, the 160 N-terminal residues of the prodomain are not included in the prodomain model. (b) Prodomain-mature domain interactions. Closeup of predicted interactions between the mature domain (magenta) and the ERFNIN (R^185^) and GNFD (E^210^; F^214^) motifs of prodomain (cyan). Blue dashed lines indicate presumed stabilizing interactions (both electrostatic as well as hydrophobic) between residues [24].

The 3D structures of the mature domain of falcipain-2 and falcipain-3 have been solved [25, 26], and the profalcipain-2 model suggests that conserved residues provide stability to the inhibitory domain (Figure 4(a)). Modeling, based on the solved structure of procathepsin B and procathepsin L [27, 28], shows that the charged pair Arg^185^ and Glu^221^ of the prodomain appear to form a salt bridge (Figure 4(b)). Further, Glu^210^ from the GNFD motif may form a separate salt bridge with Lys^403^ in the mature domain. Phe^214^ may participate in nonpolar interactions and possibly pi stacking interactions, with two tryptophan residues in the mature domain, Trp^449^ and Trp^453^. The model also suggests that the prodomain of falcipain-2 binds the mature domain in a manner similar to that of procathepsin K and L, inhibiting catalytic activity by blocking substrate access to the active site (Figure 4(a)).

### 2.3. The N-Terminal Part of the Mature Domain Is Required for Refolding

Falcipain subfamily proteases have features that are unusual for family C1A proteases. They encode short N-terminal extensions of the mature domain, which is unique among described papain family proteases [13, 14]. The N-terminal extensions allow folding of the mature domain to active enzymes [29]. Other papain family enzymes required the prodomain for proper refolding [30]. Looking at the family of C1A cysteine protease sequences on the Merops database (http://merops.sanger.ac.uk/) have identified more than 40 members with an N-terminus extension of 12 amino acids at this location. Notably, 18 of these sequences were those of falcipains and homologues from other plasmodial species. It would be interesting to find out the role of those unique extensions.

**Figure 5:**
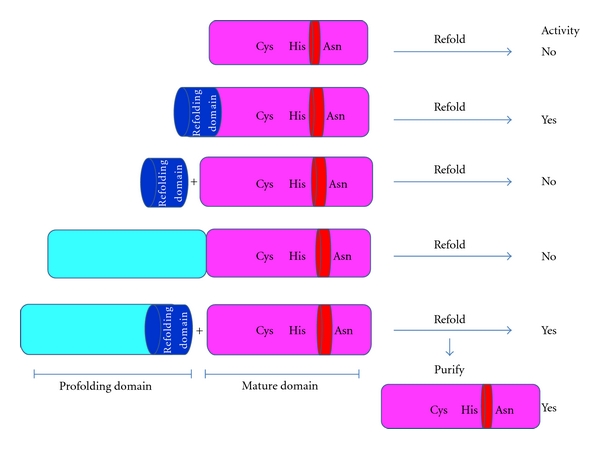
Expression and refolding of different constructs of falcipain-2. Different constructs were used to study refolding. The mature domain was expressed alone or with the refolding domain either in cis or trans. In the last construct, folding of mature domain was done in trans with profolding domain. After purification of the mature domain, the activity was assayed [29].

To better characterize the determinants of folding for falcipain-2, Pandey and Sijwali et al. expressed multiple prodomain and mature constructs of the enzyme in *E. coli* and assessed their abilities to refold by checking their activity [23, 29]. This folding study showed that refolding of the mature domain could be achieved when the mature domain was covalently joined with the N-terminus extension (Figure 5). Only 12 amino acids of the N-terminus extension (refolding domain) are necessary for refolding. Deletion of 12 amino acids from N-terminus segment of the mature domain yielded a construct incapable of correct folding, but inclusion of this segment in trans allowed folding to active falcipain-2. Correct folding also occurred when the catalytic domain was refolded with a separate prodomain-folding domain construct but not with an isolated folding domain peptide [23, 29] (Figure 5). These results indicated that the prodomain mediates the interaction between the mature and folding domain when they were not covalently bound. However, it was not clear from these studies, whether the amino-terminal extension is required only for folding or whether it also plays an essential role in mediating enzyme activity. To determine whether the folding domain was required for activity, Pandey et al. incubated the mature domain with the prodomain-folding domain in refolding buffer. After refolding and purification of the mature domain by anion exchange chromatography, the purified refolded mature domain still showed activity [29]. Together, these results suggest that the refolding domain is only required for refolding, and once refolding is accomplished, the refolding domain is not required for activity [29].

The N-terminus extensions of different falcipain subfamily enzymes have limited (20–45%) sequence identity but are functionally conserved. Chimeras of the falcipain-2 catalytic domain with the refolding domain of six other plasmodial proteases folded normally and had similar kinetic parameters to those of recombinant falcipain-2. These results showed that refolding domain can be swapped between the plasmodial proteases that harbor the same function. The above-mentioned experiment also indicates that all plasmodial cysteine proteases including falcipain-3 also use N-terminus extension as a refolding domain [29].

Structural analysis further reveals that the folding domain, in fact, has a short but significant element of secondary structure. Glutamate^120^ of the mature domain forms a buried hydrogen bond with tyrosine^13^ and a salt bridge with arginine^5^ of the refolding domain [25]. This suggests that the small folding domain, though shorter than the standard papain family prodomain, still plays a very important role in folding by stabilizing the mature domain by a hydrogen bond and a salt bridge [25]. This is analogous to a similar interaction in falcipain-2, where tryosine^12^ of the refolding domain of falcipain-3 provides hydrogen bonding anchor to glutamate^147^ of the mature domain of falcipain-3 [26]. These results indicate that the folding domains of falcipain-2 and falcipain-3 stabilize the mature domain in a similar fashion.

### 2.4. The C-Terminus Insert as a Hemoglobin Binding Domain

Falcipain-2 and 3 efficiently hydrolyze human hemoglobin in the acidic food vacuole of parasite [17, 18]. It has recently been suggested that hemoglobin hydrolysis is not a highly ordered process but rather proceeds with rapid cleavage by falcipains at multiple sites [31]. Pandey et al. showed that falcipains have a hemoglobin binding domain near the C-terminus end [32]. Falcipain subfamily proteases contain an unusual motif near the C-terminus, which is present between the highly conserved active site histidine and asparagine residues (Figure 2). A motif of identical size (14 aa) is found in all studied proteases of this subfamily of falcipain, although sequence identity is modest. In case of falcipain-2 and falcipain-3, motifs are made up of a 14 residue *β*-hairpin. The secondary structure in this region is well conserved between falcipain-2 and falcipain-3 [26]. Smaller motifs are present in the sequences encoding *P. falciparum *serine repeat antigens (10 aa) and dipeptidyl peptidase I (8 aa), and larger (20 aa) motifs are present in two other putative *P. falciparum *dipeptidyl peptidase genes [14, 33].

To evaluate the function of the unusual C-terminal motif in falcipains, a mutant enzyme was made by deleting that motif lacking 10 amino acids from falcipain-2, and the biochemical properties of wild and mutant enzymes were compared [32]. Native PAGE, Biacore, and gel filtration studies indicated that the motif mediates specific interactions with hemoglobin [32]. It has also been demonstrated by other group that falcipain-2 also interacts with hemoglobin [34]. In fact, falcipain-2 has relatively higher affinity for methemoglobin (kD is 0.8 *μ*M) than hemoglobin (kDa is 3.3 *μ*M) [34]. It had been demonstrated that several factors contribute to the formation of methemoglobin during malarial infection, including acidic pH of plasmodial food vacuole, oxidative damage in RBC [35, 36], which causes an increase in methemoglobin content to up to 20–42% in the plasmodial food vacuole. Thus, the higher affinity of falcipain-2 for methemoglobin looks like an adaptation to the specific conditions in the infected RBC. The enzyme without this motif showed negligible activity against hemoglobin or globin. Further, a peptide encoding the motif blocked hemoglobin hydrolysis but not the hydrolysis of casein, indicating that the motif specifically interacts with hemoglobin [32]. Thus, this study suggests that the motif mediates the most biologically relevant activity of falcipain, the hydrolysis of hemoglobin during trophozoite stage.

Falcipains may also have other functions in erythrocytic malaria parasites, and it would be interesting to explore the role of the motif in hydrolysis of other proteins of potential biological relevance. In the case of other members of the falcipain subfamily, it is likely that the encoded motifs share the same functions as the falcipain motif, because a number of members of this subfamily have been also shown to be hemoglobinases. The sequence conservation among the motifs of falcipain subfamily proteases is not high, but, as in the case of the N-terminal extension that mediates protein folding, the presence of these unusual motifs in all related proteases probably indicates a conserved function. For genes encoding more distantly related enzymes, including falcipain-1, dipeptidyl peptidases, and serine repeat antigens, C-terminal motifs vary greatly in sequence and size, and specific functions of predicted motifs are undefined. Data indicates that falcipain-2 captures hemoglobin via its C-terminal motif before subsequently cleaving the substrate at multiple sites. Further, the structure of falcipain indicates a protruding configuration for the motif, surrounded by a predominant negative charge [25], and it may be that charged residues are crucial for interaction with hemoglobin. It is proposed that hemoglobin, which has many charged surface residues, first binds at the motif through charge-charge interaction and brings hemoglobin closer to the active site before hydrolysis. The function of the motif in mediating catalysis despite its separation from the active site somewhat resembles that of the hemopexin domain of matrix metalloproteases. The hemopexin domain is required for both the efficient cleavage of collagen [37] and the formation of complexes with both inhibitors and proteases to mediate biological activities [38]. Thus, as is the case for the falcipain-2 motif, the hemopexin domain serves to facilitate biologically relevant protein-substrate and protein-inhibitor interactions. However, the hemopexin domain is approximately 20 times larger than the falcipain motif and, unlike the motif, is separated from the catalytic domain by a hinge region. Therefore, it appears that falcipains and matrix metalloproteases use similar means of biological control, but the specific mechanisms by which the proteases mediate interactions with substrates and inhibitors are different.

Another papain family protease, cathepsin K, forms a pentameric complex with chondroitin sulfate to facilitate hydrolysis of collagen [39]. Having analogy to falcipain-2-hemoglobin interaction, structural requirements for complex formation between cathepsin K and chondroitin sulfate are also required for hydrolysis of the biological substrate, collagen. It is interesting to raise a question, why do falcipains contain a unique motif that mediates the hydrolysis of hemoglobin? This mechanism might not have evolved simply to facilitate hemoglobin hydrolysis, because other papain family enzymes without C-terminus motifs still can hydrolyze hemoglobin. It looks like that the utilization of a specific motif at this region to mediate enzyme-substrate interaction is an unusual but conserved feature of plasmodial cysteine proteases. It may be possible that an evolutionary bottleneck occurred in ancestral plasmodial cysteine proteases, and those enzymes might not be that efficient in hemoglobin hydrolysis. In addition, plasmodial cysteine proteases might have evolved with introduction of specific motif for efficient hemoglobin hydrolysis.

The interaction of a major hemoglobinase, falcipain-2, with most biologically relevant substrate, hemoglobin, could be an interesting starting point for the development of an effective drug. The structure of falcipain-2 hemoglobin binding domain will guide the design of inhibitors that should interfere with hemoglobin binding.

## 3. Structural Basis of Inhibition of Falcipains

### 3.1. Inhibition of Falcipains by Macromolecules

There are two major classes of cysteine protease inhibitor, small inhibitors like leupeptin, vinyl sulfones, E64, and another class known as macromolecular inhibitor. Macromolecule inhibitors are polypeptide in nature, which are generally present inside the organisms. These endogenous cysteine protease inhibitors have been described in a number of eukaryotic systems. Here, we will discuss three major cysteine protease inhibitors, cystatin, chagasin, and falstatin.

#### 3.1.1. Inhibition of Falcipains by Cystatin

**Figure 6:**
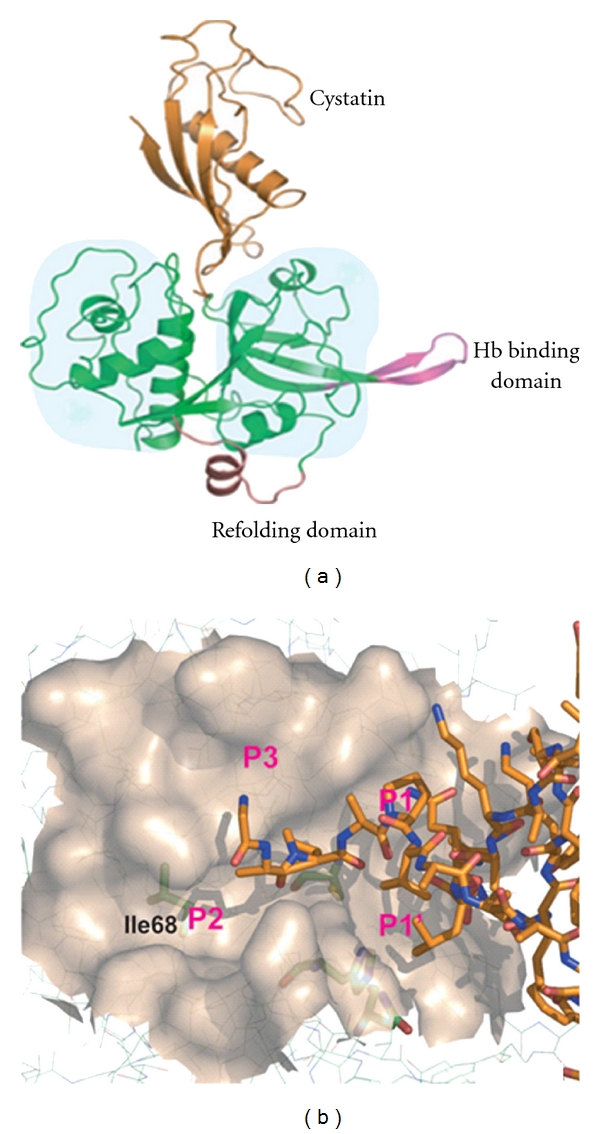
(a) 3D structure of falcipain-2-cystatin complex. Cystatin is colored in orange, and falcipain-2 protease is colored in green. Refolding domain and hemoglobin binding domain highlighted in pink and salmon, respectively. The shaded area in the figure corresponds to the size of human cathepsin H, superimposed on falcipain-2 in light blue. (b) The catalytic Cys, His, and Asn are depicted in stick mode in green, and substrate binding pockets are labeled P3 to P1′. Ile-68 at P2 is depicted in stick mode to illustrate a slight protrusion in the S2 pocket of falcipain-2.

Excluding the two unique motifs discussed earlier, the rest of the falcipains are structurally similar to homologous proteases in the papain family. Wang et al. studied the falcipain-cystatin interactions by solving the structure of this complex [25]. Cystatin was used as a falcipain inhibitor with the expectation that protein-protein interaction over large surfaces would enhance crystal quality and thus facilitate structural determination. Cystatins inhibit a wide range of papain family cysteine proteases with high affinity making them ideal candidates for cocrystallization with falcipain. Falcipain and chicken egg white cystatin formed 1 : 1 complex (Figure 6(a)). The inhibitory constants (Ki) of cystatin for falcipain-2 and falcipain-3 are 6.5 nM and 100 nM, respectively [25]. It is interesting that cystatin is a more potent inhibitor of falcipain-2 than falcipain-3, which suggests that cystatin regulates both the falcipains with different rates. This might be important biologically, since their timing of expression is slightly different. The falcipain-cystatin complex shares many features with two known cysteine protease-cystatin structures: cathepsin H-stefin A and papain-stefin B (the stefins are a subclass within the cystatin superfamily [41, 42]). These cystatins bind to target proteases in similar orientations, resulting in extensive interactions with the target proteases (Figure 6(b)). Cystatin largely interacts with falcipain-2 at prime sites where substrate-binding pockets are relatively shallow and less defined, but falcipains also have binding specificity at nonprimed sites. Although, the binding preference is not as pronounced as that found at the S2 pocket (Figure 6(b)). Cystatin is only able to access the solvent exposed periphery of the nonprime site of falcipain-2 and majority of its binding occurs at the prime end of the active site.

Unlike most other cathepsin L-like cysteine proteases, falcipain-2 strongly prefers a Leu at P2 over a Phe [43]. Crystal structures indicate that this preference may be due to Ile^68^ in falcipains, because it creates a small protrusion in the otherwise flat base of the S2 pocket, which restricts aromatic side-chain binding [25] (Figure 6(b)). The falcipain–cystatin complex can be exploited to design potent inhibitors of the malaria parasite. A recent study indicates that a peptide based on cystatin binding residues blocked the activity of falcipain-2 and led to accumulation of undegraded hemoglobin in the food vacuole [44].

#### 3.1.2. Inhibition of Falcipain by Chagasin

Chagasin is a cysteine protease inhibitor that was first identified in *Trypanosoma cruzi* as the physiological regulator of cruzain, the major cysteine protease [45, 46]. Cruzain is also a papain-like (Clan CA) cysteine protease that is expressed in all stages of *T. cruzi* life cycle. It is a key virulence factor of *T. cruzi*, the infectious agent responsible for the leading cause of heart disease in Latin America, called the Chagas disease [47].

**Figure 7:**
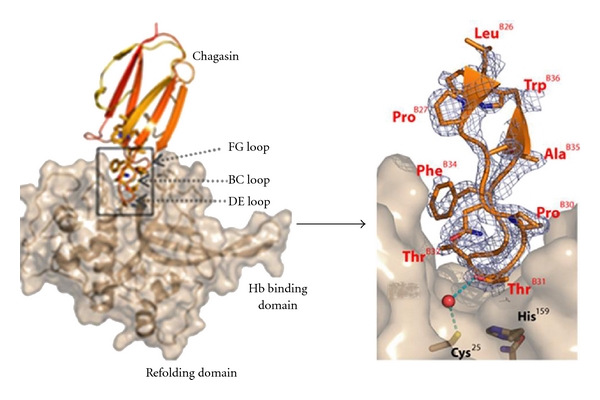
Structure of falcipain-2-chagasin complex: overall structure of chagasin with falcipain-2, chagasin in red and falcipain-2 in gold. Key binding interactions between chagasin and falcipain-2 can be seen [40].

Chagasin is associated with cruzain during its trafficking to specific compartments of the parasite cell, and accumulated evidence suggests that the primary role of chagasin is in posttranslational regulation of protease activity [45]. Chagasin is also a potent inhibitor of falcipains and demonstrates 1 : 1 binding with falcipain-2 (Figure 7). The protease-binding loops (BC, DE, and FG) in chagasin form a well-aligned wedge that fills the active site groove of falcipain-2 to obstruct substrate binding (Figure 7) [40]. The BC loop is one of the three signature motifs that contribute to the inhibition of the cysteine protease. This is confirmed by a synthetic peptide corresponding to the BC loop of the chagasin like protein from *E. histolytica, *which specifically blocked the activity of cysteine proteases [49]. It is noteworthy that Thr 31 in the BC loop of chagasin binds to the catalytic Cys at the falcipain-2 active site by water-mediated hydrogen bonds (Figure 7). It is likely that the highly conserved Thr serves as a key functional residue among chagasin-like inhibitors. Another interesting feature of the falcipain-2-chagasin interaction is the highly mobile DE loop like in E64, a strong irreversible inhibitor of cysteine protease, which occupies the nonprime site. In summary, the BC loop, mobile DE loop, and the RPW/F motif in the FG loop are the key elements for binding with falcipain-2.

#### 3.1.3. Inhibition of Falcipain by Falstatin

Disturbance of the equilibrium between cysteine proteases and natural inhibitors is a key event in the pathogenesis of cancer, rheumatoid arthritis, osteoporosis, and emphysema. In case of malaria, falstatin has been recognized as an endogenous cysteine protease inhibitor in *P. falciparum*. Erythrocytic *P. falciparum* parasites express falstatin, a potent inhibitor of falcipains and many other cysteine proteases [50]. But it is unknown how falstatin regulate the *P. falciparum* cysteine proteases. The stage-specificity of falstatin expression and the effects of anti-falstatin antibodies on parasite development suggest that this inhibitor facilitates a process that also requires proteolytic activity, the invasion of erythrocytes by *P. falciparum* merozoites. Falstatin is a competitive and reversible inhibitor of falcipains, as demonstrated by increasing calculated *K*_*m*_ values but similar *V*_max⁡_ values with increasing concentrations of falstatin [50].

To evaluate the mechanism of inhibition of cysteine proteases by falstatin, Pandey et al. tested the ability of active site-inhibited falcipain-2 to compete with active falcipain-2 for binding with falstatin. In contrast to results with the prodomain, the inhibitory effect of falstatin was not affected by the presence of active site inhibited falcipain-2 [50]. Thus, the binding of falstatin to falcipain-2 appears to be via interaction with the enzyme active site.

### 3.2. Inhibition of Falcipains by Small Molecule Inhibitors (Leupeptin, E-64, and Vinyl Sulfones)

**Figure 8:**
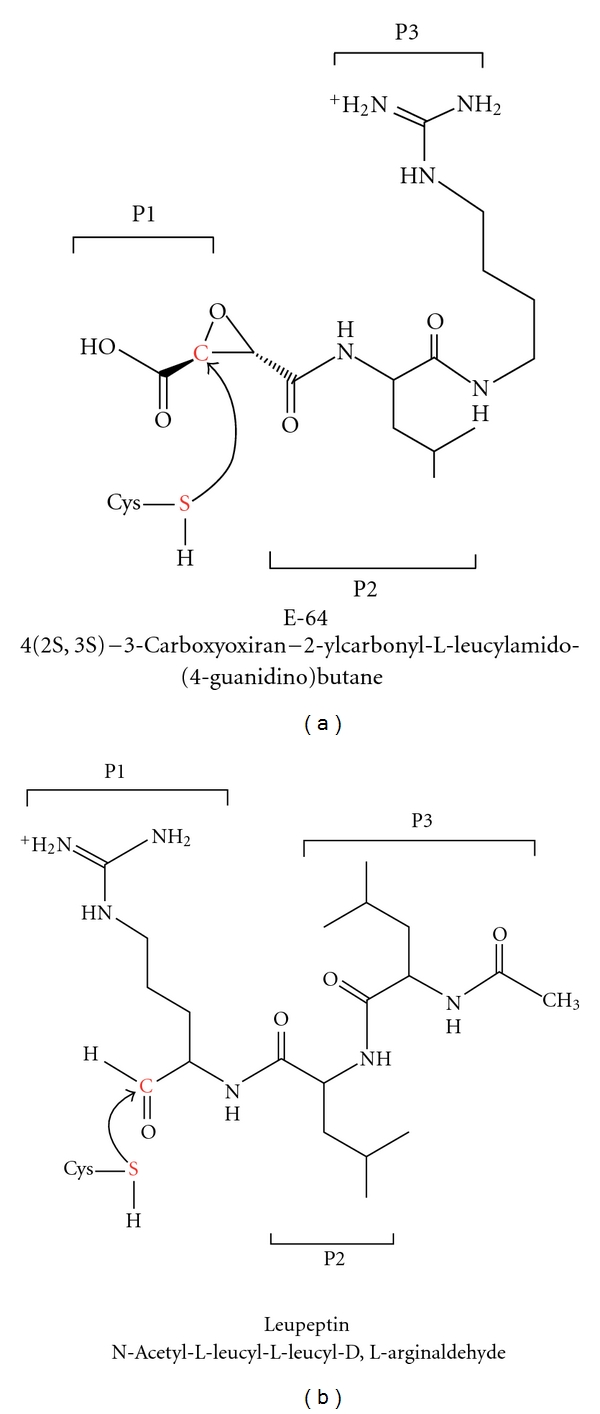
Chemical structures of E-64 and leupeptin. The positions of P1, P2, P3 of the inhibitors that occupy the S1, S2, and S3 subsites of enzyme, respectively, are labeled. Inhibitor and enzyme groups which are involved in covalent bond formation are shown in red.

Leupeptin, E-64, and vinyl sulfones are major cysteine protease inhibitors that bind to the active site [26, 48]. The active sites of both enzymes are located in a cleft between the structurally distinct domain of the papain-like fold. The structures of falcipain-2 and falcipain-3 have been determined in complex with these small inhibitors. E-64, and leupeptin interact with residues in the S1, S2, and S3 subsites of falcipain-2 and falcipain-3, corresponding to the P1, and P2, and P3 position of the inhibitors (Figure 8).

**Figure 9:**
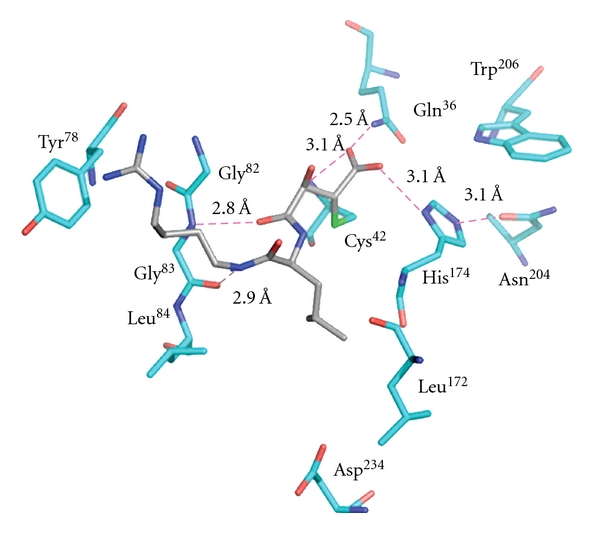
Interaction between falcipain-2 and E-64. The residues in the active site of falcipain-2 are colored blue and labeled, and E-64 is colored in grey. The interactions with enzyme and inhibitor are in pink [26].

The conserved catalytic residues of falcipain-2 and falcipain-3 (Gln ^36/45^, Cys ^42/51^, His ^174/183^, Asn ^204/213^, resp.) are similarly oriented with respect to the co-crystallized inhibitors. Inhibitors display binding modes with their partner enzymes similar to those found in other papain family enzymes [26]. The inhibitors bind to the main chain of falcipain-2 and falcipain-3 by glycine (Gly^83^ in falcipain-2 and Gly^92^ in falcipain-3) residue that is highly conserved in the S3 subsite of clan CA cysteine proteases (Figure 9). In cocrystallization, these residues form hydrogen bonds with the O and N atoms of the inhibitor backbone. In the case of the falcipain-2 complex with E-64, enzyme active sites Gln^36^, Ser^41^, Cys^42^, Asn^81^, and His^174^ are involved in the formation of additional hydrogen bonds with E-64. In the falcipain-3-leupeptin complex, Gln^45^, Cys^51^, and Asn^182^ also act as hydrogen bonding partners to the inhibitor. The enzyme-inhibitor complex stabilize by a series of possible hydrophobic interaction using nonpolar region of Gly^40^, Tyr^78^, Gly^82^, leu^84^, Ser^149^, Leu^172^, Asn^173^, and Ala^175^ in falcipain-2, and Tyro^90^, Gly^91^, Tyr^93^, Ile^94^, and Ser^158^ in case of falcipain-3. The active site cysteine of falcipain-2 forms a covalent, irreversible hemithioketal with the E-64 epoxy carbon (Figure 9) [26].

**Figure 10:**
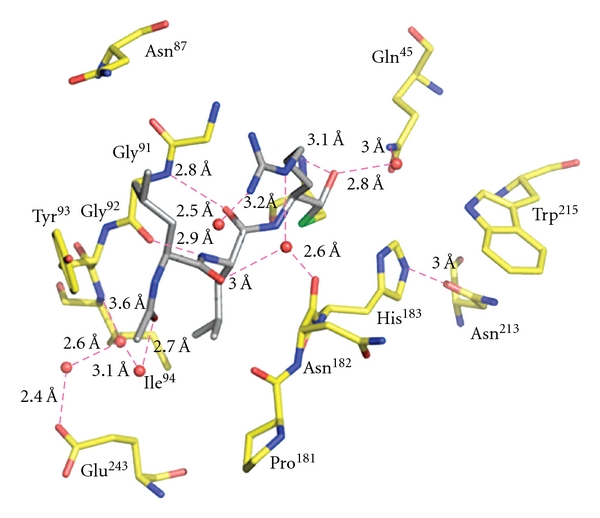
Interaction between falcipain 3-leupeptin: the residues in the active site of falcipain-3 are colored yellow and labeled, and leupeptin is colored in gray. The interactions with enzyme and inhibitor are in pink [26].

The active site cysteine of falcipain-3 forms a covalent, reversible hemithioacetal with the asymmetric carbonyl carbon of leupeptin (Figure 10) [26]. The interaction pattern of E-64 and leupeptin with all papain family enzymes is conserved. The carboxyl group of E-64 and carbonyl group of leupeptin occupy the oxyanion hole formed by the conserved catalytic residues. Falcipain-2 and Falcipain-3 have a clear preference for substrates/inhibitors that contains a leucine at P2 position, and both leupeptin and E64 contain a Leu at P2. Surprisingly, falcipain-3 has been shown to be much less efficient at hydrolyzing peptide substrates and more difficult to inhibit with small peptidyl-based inhibitors compared with falcipain-2 [16, 51].

The structure that forms the active site is highly conserved between falcipain-2 and falcipain-3, and there is no notable movement in the peptide backbone or loop region. However, superimposition of falcipain-2 and falcipain-3 structures highlights two important substitutions in the S2 subsite. Falcipain-2 has Asp^234^ and Leu^84^, and falcipain-3 has Glu^243^ and Tyr^93^ [26]. This position of the S2 subsite is known to be a key determiner of the specificity (Figures 9 and 10). In comparison to Asp^234^ in falcipain-2, the additional side chain carbon in Glu^243^ of falcipain-3, this increased the size as well as flexibility at this position. In case of falcipain-3–leupeptin complex, the larger and more flexible residue (Glu^243^) at the bottom of S2 subsite and bulkier residue (Try^93^) create a narrow wall of the S2 subsite [26], as compared to the falcipain–E-64 complex (Figure 10). The overall structures of falcipain-3–leupeptin and falcipain-2–E-64 complexes show a similar mode of binding and inhibition compared with macromolecule inhibitors like chagasin and cystatin.

**Figure 11:**
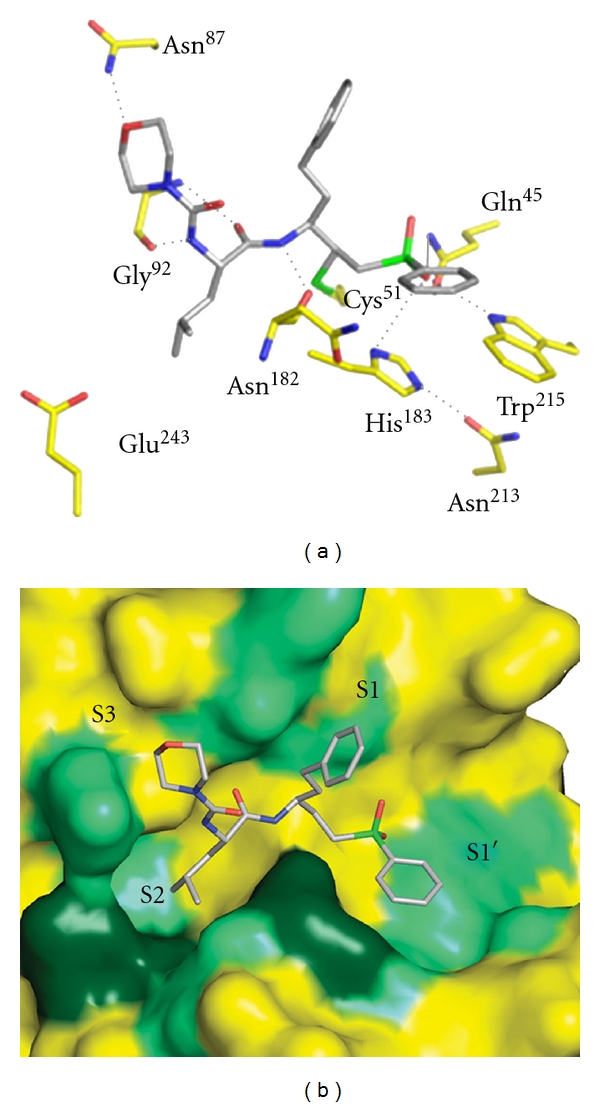
(a) Interaction of falcipain-3 with morpholinourea-leucine-homophenylanyl-phenyl vinylsulfone (Mu-Leu-Hph-VsPh). The residues in the active site of falcipain-3 are colored yellow and labeled, and morpholinourea-leucine-homophenylanyl-phenyl vinylsulfone is colored gray. The interactions with enzyme and inhibitor are in black dots. (b) The active site of falcipain-3 with hydrophobic environment. The inhibitor occupies the S1′, S1, S2, S3 subsites of enzyme. Hydrophobic residues are colored light green, and polar residues that interact with the inhibitor by nonpolar C–C bonds are colored dark green [48].

Like E64 and leupeptin, vinyl sulphones (Mu-leu-Hph-VsPh) have been shown to be effective inhibitors of a number of papain-family-like cysteine proteases. Mu-Leu-Hph-VSPh is a potent irreversible inhibitor of falcipain-2 and falcipain-3 [48]. The cocrystallization of falcipain-3 with Mu-Leu-Hph-VsPh indicates that inhibitor binds the respective S1 and S3 subsites to form an irreversible, covalent bond with the sulfur of the active site cysteine thiol in enzyme [48] (Figure 11). Falcipain-3 prefers Leu at P2 of Mu-Leu-Hph-VsPh. Given the hydrophobic nature of the P1 and P2 substituents in Mu-leu-Hph-VsPh, the active site of falcipain-3 is lined with a number of residues that are able to make nonpolar contacts with their respective inhibitor [48] (Figure 11).

As seen in the crystal structures of falcipain-3 with the above inhibitor, the residue at the bottom of the S2 subsite (Glu^243^) points out of the pocket to avoid a potentially unfavorable interaction with the bulky Phe residue at the P2 position of inhibitor. Cysteine proteases of the malaria parasite may be targeted for inhibition by vinyl sulphones. Indeed, vinyl sulphones-based cysteine protease inhibitors are already being used in preclinical trials for the Chagas disease [52].

## 4. Summary and Conclusion

The understanding of the cysteine proteases of malaria parasites has increased markedly in recent years. Since cysteine proteases that play an important role in the parasite life cycle by degrading erythrocyte proteins, most notably hemoglobin, are attractive targets for antimalarial chemotherapy. Falcipain-2 and falcipain-3 are the best characterized cysteine proteases of malaria parasite, the structure and function of different domains and their interaction with small and macromolecular inhibitors are studied. The structure-function study of falcipains and interaction with inhibitors will provide detail insights to develop rational design of inhibitor against falcipains. Structure-guided approaches should have great role in the design of potent and highly selective inhibitor. Efforts to optimize current inhibitors based on the structure-function of falcipains are currently underway.
